# First example of organocatalysis by cathodic N-heterocyclic carbene generation and accumulation using a divided electrochemical flow cell

**DOI:** 10.3762/bjoc.18.98

**Published:** 2022-08-05

**Authors:** Daniele Rocco, Ana A Folgueiras-Amador, Richard C D Brown, Marta Feroci

**Affiliations:** 1 Department of Ingegneria Meccanica ed Aerospaziale, Sapienza University, via Eudossiana, 18, 00184, Rome, Italy; 2 School of Chemistry, University of Southampton, Southampton SO17 1BJ, UK; 3 Department of Scienze di Base e Applicate per l’Ingegneria, Sapienza University, via del Castro Laurenziano, 7, 00161, Rome, Italy

**Keywords:** Breslow intermediate, cathodic reduction, flow electrochemistry, N-heterocyclic carbene, oxidative esterification

## Abstract

In this paper we present the first electrochemical generation of NHC carried out in a divided flow cell. The flow cell operated in the recycle mode. The need for a divided cell derived from the anodic electroactivity of the electrogenerated carbene. In order to have NHC accumulation in the catholyte, the Nafion membrane (cell separator) was pretreated with an alkaline solution. The formation of NHC was quantified as its reaction product with elemental sulfur. The NHC was successfully used as organocatalyst in two classical umpolung reactions of cinnamaldehyde: its cyclodimerization and its oxidative esterification.

## Introduction

Ionic liquids (ILs) are well known salts, at present used in a wide variety of chemical fields. The first definition of ionic liquids was given by Paul Walden in 1914: “*they are materials composed of cations and anions, that melt around 100 °C or below as an arbitrary temperature limit”* [1]. ILs are salts formed by non or weakly coordinated cations and anions, usually bulky organic cations and inorganic or organic anions such as ^−^BF_4_, ^−^PF_6_, ^−^N(CF_3_SO_2_)_2_, etc. [2–3].

The physicochemical properties of RTILs (room temperature ionic liquids) are reported in the literature and include very low vapor pressure, and thus the possibility to recycle them, good stability, low flammability, good solubility ability for many molecules, salts and gases, the possibility to use them as reagents and/or solvents, large electrochemical window, good electrical conductivity, their ionic nature sometimes increases the reactivity and the selectivity of reactions and usually the product separation is easy [4–5]. Among ILs, imidazolium derivatives are the most studied, in part due to their ease of synthesis, low cost and diverse applications from solvents and reagents in synthesis, to supporting electrolytes in electrochemistry [6].

**Scheme 1:**
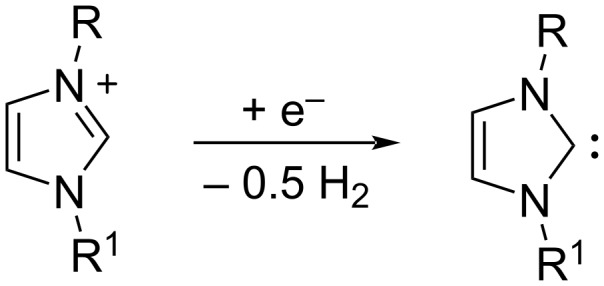
Electrochemical generation of NHC.

The imidazolium cation can be modified by the presence of a base or by a single electron cathodic reduction of the C–H between nitrogen atoms of the imidazolium ring (Scheme 1), inducing the formation of a N-heterocyclic carbene (NHC) [7–8]. In recent years, NHCs have achieved great success: they have been frequently used as ligands in organometallic catalysts [9] and as versatile organocatalysts [10] in a very wide range of organic reactions such as classical benzoin condensation, transesterification, acylation, Knoevenagel reaction, Claisen condensation etc.

**Scheme 2:**
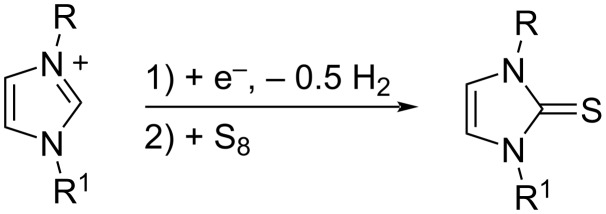
Transformation of electrochemically generated NHC into the corresponding thione by its reaction with elemental sulfur.

The electrochemical generation of carbenes from ILs avoids the use of strong bases and the formation of byproducts (dimers of carbenes and nitrogen dealkylation products), where the IL acts as NHC precursor, solvent, and supporting electrolyte, needing no additional chemicals in the electrolytic cell [11]. An added attraction of this approach is that unstable NHCs are generated in situ, where they may be used as basic or nucleophilic species. Due to the difficulty isolating highly reactive NHCs, the concentration of the obtained NHC solution can be determined indirectly by addition of elemental sulfur after the electrolysis, which realizes quantitative conversion to the corresponding thione (Scheme 2) [12].

**Scheme 3:**
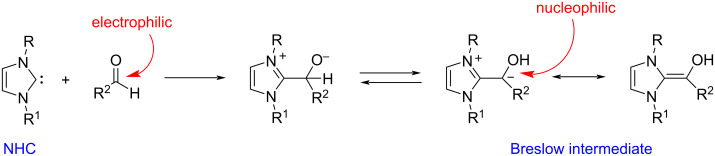
Umpolung of the aldehyde carbonyl carbon atom. Formation of the Breslow intermediate using NHCs.

NHCs are used as organocatalysts in many reactions of aldehydes (mainly aromatic) [13–14]. In fact, the reaction of NHCs with aldehydes can lead to the formation of the “Breslow intermediate” [15], in which the reactive character of the carbonyl carbon atom is reversed (umpolung) from electrophilic to nucleophilic (Scheme 3).

This approach can be exploited in many organic reactions, such as: the benzoin condensation [16–17], esterification and amidation of benzaldehydes and cinnamaldehydes [18–19], synthesis of γ-butyrolactones [20], synthesis of 1,3-diketones [21], etc.

Electrosynthesis is considered as a more sustainable approach to perform chemical reactions, and an interesting alternative to conventional synthetic methods both in laboratory and industry processes. In fact, the electron may be considered to be a clean reagent, which replaces toxic chemical redox reagents and dangerous procedures [22–25]. At present, electrosynthesis in batch is more widely used and reported in literature, but some disadvantages can be encountered: the need for high concentrations of supporting electrolyte, poor performance for synthesis such as slow rates of conversion, low selectivity and reproducibility [26]. As a matter of fact, these problems can be addressed by using flow electrochemistry, usually achieving higher rates of conversion of reagents to products [27]. Moreover, electrochemical flow cells can have a very small gap between the electrodes so that lower concentrations of supporting electrolytes are needed to provide sufficient conductivity [28]. Applications of flow electrochemistry reported in the literature are mainly devoted to anodic oxidations, carried out in undivided cells, in which the counter electrode reaction at the cathode is usually H_2_ evolution [29]. The use of divided cells is less common in organic electrosynthesis, mainly due to complications inherent with membranes. Useful cathodic processes are less exploited in organic electrochemistry. In the context of NHC organocatalysis in flow electrochemistry, NHC instability (and anodic electroactivity) prevented its cathodic generation and subsequent use as catalyst or reagent. Instead, the NHC was generated by chemical deprotonation using a strong base (DBU) and then applied in anodic esterification [30–32], and amidation of aromatic aldehydes [33]. Flow electrochemistry was applied to oxidize the Breslow intermediate to the corresponding electrophilic acylthiazolium intermediate, which then functioned as an acyl-transfer reagent, reacting with alcohols or amines. To the best of our knowledge, only one research group reported the cathodic reduction of an imidazolium cation to NHC, in an undivided cell under flow conditions, coupled with the anodic generation of Cu(I) from a sacrificial anode to yield the corresponding N-heterocyclic carbene complex [34–35]. In this case, irreversible capture of the NHC by the metallic cation prevented NHC oxidation/degradation.

In this paper we describe the cathodic generation and accumulation of NHC in a divided flow cell and its subsequent use as organocatalyst in the self-annulation of cinnamaldehyde and in the esterification of cinnamaldehyde.

## Results and Discussion

### Cathodic NHC generation and accumulation using a divided flow electrochemical cell. Quantification of NHC in IL solution

**Figure 1:**
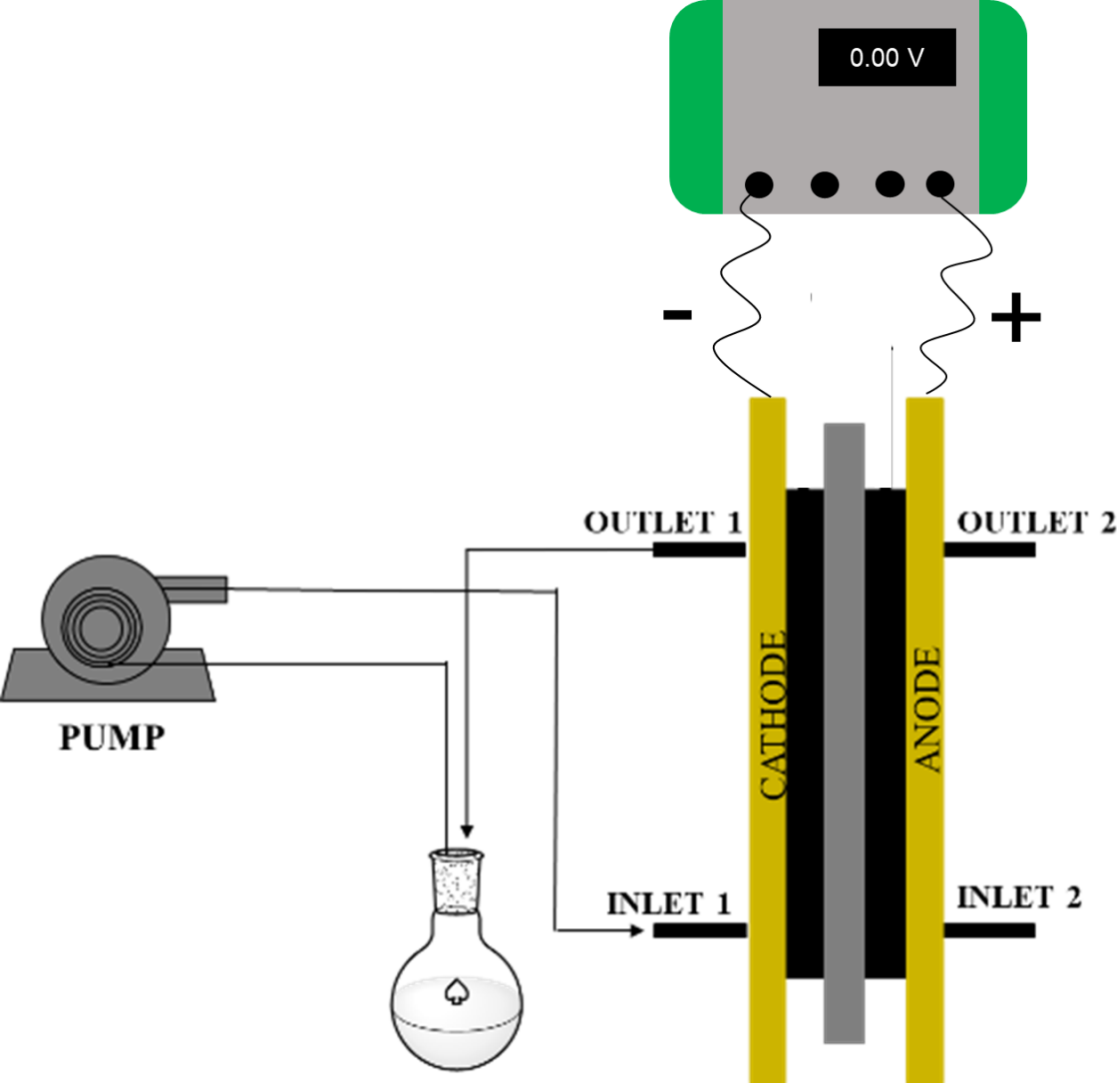
Schematic representation of a plane-parallel plate flow electrochemical reactor.

The flow cell used in this work was previously described [36]. It is based on two electrode plates separated by a spacer/gasket fabricated from an PTFE sheet with its center cut away to form the electrolyte flow chamber (Figure 1). The requirement for a divided cell (a more complicated device than the undivided configuration) arises from the need to protect electrogenerated NHC from its anodic oxidation in the absence of a consumable anode.

To ensure good sealing of the electrolysis cell, the sandwich-type arrangement of cell components was compressed between two end plates using a series of bolts. This design incorporated a solution inlet and a solution outlet for the chamber to allow uniform flow over the surface of the electrodes. In the present work, where a divided cell configuration was required, a proton-permeable membrane (Nafion^®^ 438) was inserted to separate the cathode and anode chambers, and a spacer was used on each compartment [36].

The initial goal of this work was to demonstrate the possibility to achieve NHC formation starting from 1-butyl-3-methylimidazolium tetrafluoroborate (BMImBF_4_), by cathodic reduction in a divided cell using flow electrochemistry technique, and to compare the results with the corresponding batch process. Once established, the flow electrochemistry NHC synthesis would be combined with applications as an organocatalyst in some organic transformations of cinnamaldehyde.

Firstly, the conditions for electrogeneration of the carbene in flow were optimized, quantifying the amount of NHC by means of its reaction with elemental sulfur. All the experiments were carried out using a 0.1 M solution of BMImBF_4_ in acetonitrile as catholyte in a divided cell using Nafion^®^ 438 membrane, under N_2_ atmosphere, at room temperature, under galvanostatic conditions (*I* = 134 mA) with continuous flow rate of 36 mL/min, studying the effect of cathode material, anode solution and number of Faradays per mole of IL supplied (Table 1). At the end of the electrolysis, excess elemental sulfur was added to the catholyte and the mixture was left under ultrasound irradiation for 30 minutes. The solvent was removed under reduced pressure and the residue was purified by column chromatography on silica gel.

**Figure 2:**
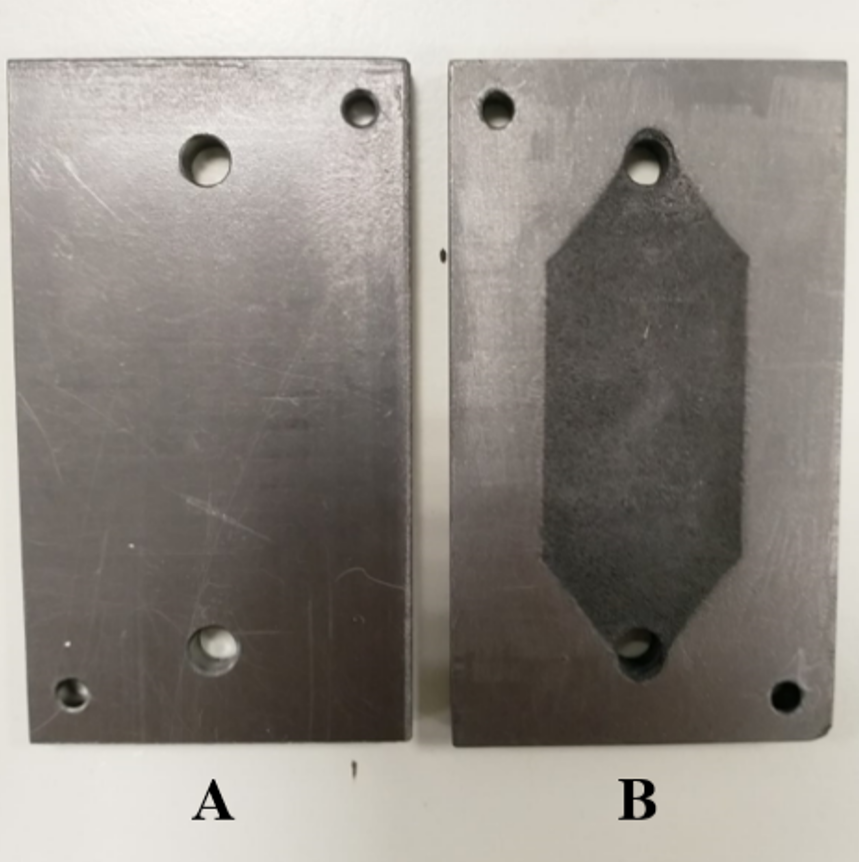
C/PVDF anode before (A) and after (B) the first experiment (Table 1, entry 1).

The first experiment (Table 1, entry 1) was carried out using stainless steel as cathode and a carbon-filled polyvinylidene fluoride (C/PVDF) plate as anode. BMImBF_4_ 0.1 M in acetonitrile was the catholyte, while the anolyte was a solution of tetraethylammonium tetrafluoroborate (Et_4_NBF_4_) in acetonitrile; after only 12 minutes of electrolysis the current flow stopped. Moreover, a consumption of anode was observed (Figure 2). It is possible that, in the absence of a suitable counter electrode process, the tetrafluoroborate anion was itself oxidized [7] leading to erosion of the anode with the formation of fluorocarbons.

**Table 1:** Electrochemical reduction of BMImBF_4_,^a^ followed by the addition of elemental sulfur^b^. Flow cell, in recycling mode.

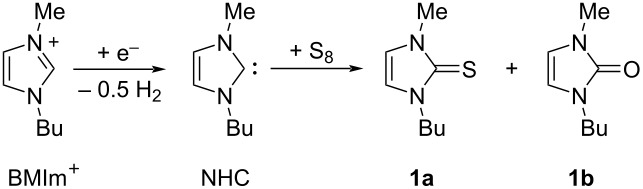						

Entry	Cathode material	Anolyte	Q (F)	Time (min)	Yield **1a**^c^	Notes

1	SS^d^	MeCN/Et_4_NBF_4_ 0.05 M, 25 mL	0.5	12	<5%	anode erosion
2	SS^d^	MeCN–MeOH (9:1)/Et_4_NBF_4_ 0.05 M, 25 mL	1.0	24	13%	–
3	Ag	MeCN–MeOH (9:1)/Et_4_NBF_4_ 0.05 M, 25 mL	1.0	24	–	–
4	Ni	MeCN–MeOH (9:1)/Et_4_NBF_4_ 0.05 M, 25 mL	1.0	24	13%	–
5	SS^d^	MeCN–MeOH (9.5:0.5)/Et_4_NBF_4_ 0.1 M, 20 mL	1.0	24	21%	Nafion^®^ alkaline pretreatment
6	SS^d^	Dry MeCN–MeOH (9.5:0.5)/Et_4_NBF_4_ 0.1 M, 20 mL	1.0	24	32% (**1b** <5%)	Nafion^®^ alkaline pretreatment

^a^Divided cell, carbon-filled polyvinylidene fluoride (C/PVDF) anode material, Nafion^®^ 438 membrane separator, room temperature, N_2_ atmosphere, galvanostatic conditions (134 mA), catholyte: BMImBF_4_/MeCN 0.1 M, 20 mL (2 mmol BMImBF_4_); flow rate: 36 mL/min. ^b^Excess S_8_ (2 mmol) added at the end of the electrolysis to the catholyte. Then energy was supplied to the catholyte (ultrasound irradiation, 35 W) for 30 minutes. ^c^Isolated yields, based on starting IL (BMImBF_4_). ^d^Stainless steel.

In order to avoid anode consumption/passivation, an alternative to oxidation of BF_4_^−^ was required as the counter electrode reaction, and 10% of methanol was added to the anolyte solution. In this case (Table 1, entry 2) 13% of thione was obtained and the electrode did not show any signs of consumption. The same yield (13%) was obtained using nickel as cathode material (Table 1, entry 4). However, using a silver electrode the corresponding thione was not observed (Table 1, entry 3) [37]. In view of the acidic character of the perfluorosulfonic acid Nafion^®^ membrane, and possible NHC protonation to form the imidazolium cation, the Nafion^®^ membrane was pretreated with an alkaline solution for 24 hours. Using stainless steel as cathode and a lower amount of methanol at the anode the quantity of thione increased to 21% (Table 1, entry 5). Under the same conditions, but using dry acetonitrile, the best yield was obtained (32%, Table 1, entry 6); in this case a small amount of imidazolone **1b** (<5%), probably due to the reaction with adventitious oxygen, was observed from the NMR spectrum. It should be underlined that in these electrolyzes BMImBF_4_ acts as both electroactive species and supporting electrolyte, and although the cathodic reduction of the imidazolium cation is a monoelectronic process, in a similar process carried out in batch the current yield usually does not exceed 50%, thus rendering necessary an excess of current [38]. The yields reported in Table 1 (entries 2 to 6) are obtained using a stoichiometric amount of electricity (1 electron per imidazolium cation). Therefore, the 32% chemical yield (with respect to starting IL) is comparable with the yield of the batch process.

### Electrogenerated NHC organocatalysis. Dimerization of *trans*-cinnamaldehyde: synthesis of 4-phenyl-5-styryldihydrofuran-2(3*H*)-one

**Scheme 4:**
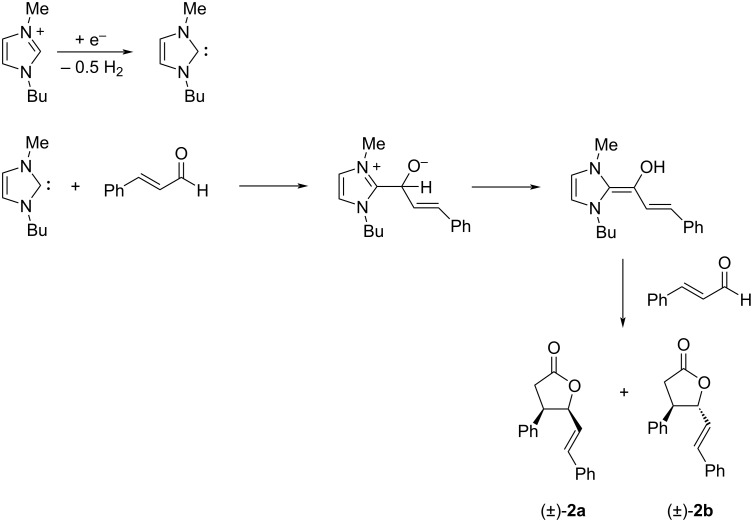
Electrogenerated NHC-catalyzed self-annulation of cinnamaldehyde.

Having demonstrated that NHCs could be generated and accumulated in a continuous flow electrochemical process, the flow methodology was applied to the self-annulation of cinnamaldehyde, a classical NHC-catalyzed reaction (Scheme 4).

All the experiments were carried out using a solution of 0.1 M BMImBF_4_ in acetonitrile (20 mL) as catholyte, stainless steel as cathode, C/PVDF as anode, in a divided cell, under N_2_ atmosphere, at room temperature, under galvanostatic conditions (*I* = 134 mA) with a flow rate of 36 mL/min, changing anode solution and number of Faradays per mole supplied (Table 2). Following the experimental results previously obtained, the Nafion^®^ membrane was always pretreated with an alkaline solution. At the end of the electrolysis, 1 mmol of cinnamaldehyde was added to the catholyte and the mixture was left under stirring for two hours at room temperature. Workup and column chromatography yielded a diastereomeric mixture of γ-butyrolactones **2a** and **2b**.

**Table 2:** Electrochemical synthesis of γ-butyrolactones **2a** and **2b** by conjugate umpolung reaction.^a^

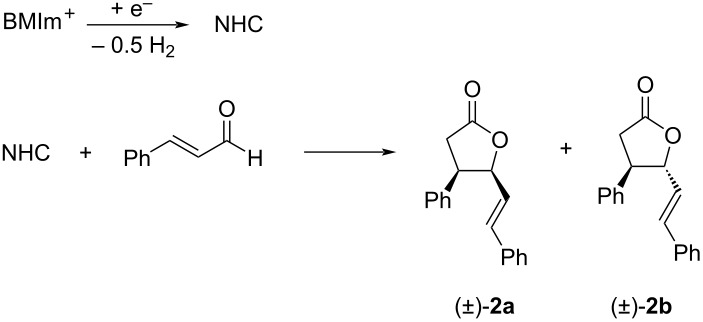					

Entry	Anolyte	*Q* (F)^b^	Time (min)	Yield^c^	

				**2a**	**2b**

1	MeCN–MeOH (95:5)/Et_4_NBF_4_ (0.1 M), 20 mL	0.50	6	15%	8%
2	MeCN–MeOH (95:5)/Et_4_NBF_4_ (0.1 M), 20 mL	1.00	12	43%	24%
3	MeCN–MeOH (95:5)/Et_4_NBF_4_ (0.1 M), 20 mL	2.00	24	53%	26%
4	MeCN–MeOH (98:2)/Et_4_NBF_4_ (0.1 M), 20 mL	1.00	12	42%	32%
5	MeCN–DMSO (95:5)/Et_4_NBF_4_ (0.1 M), 20 mL	0.50	6	7%	<5%
6	MeCN–DMSO (95:5)/Et_4_NBF_4_ (0.1 M), 20 mL	1.00	12	20%	12%

^a^Divided cell, carbon-filled polyvinylidene fluoride (C/PVDF) anode, stainless steel cathode, alkaline pretreated Nafion^®^ 438 membrane as compartments separator, room temperature, N_2_ atmosphere, galvanostatic conditions (134 mA), catholyte: BMImBF_4_/MeCN 0.1 M, 20 mL (2 mmol BMImBF_4_); flow rate: 36 mL/min; 1 mmol of cinnamaldehyde added at the end of the electrolysis to the catholyte. ^b^With respect to the starting BMImBF_4_. ^c^Isolated yields, based on starting cinnamaldehyde.

**Scheme 5:**
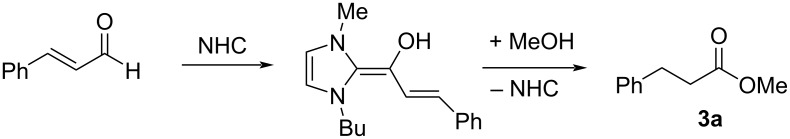
Byproduct obtained from the reaction between methanol and the Breslow intermediate.

Table 2 reports the results obtained by changing the anolyte composition and the amount of applied electricity. In all cases, with regards to the *trans*/*cis* diastereomeric ratio, we observed that the *cis* isomer **2a** was predominantly formed. The diastereoselectivity was not high; however, *trans* and *cis* γ-butyrolactones were easily separated by column chromatography. Increasing the applied charge from 0.5 F (Table 2, entry 1) to 1.0 F (Table 2, entry 2) improved the yield of both *cis* (**2a**) and *trans* (**2b**) lactones by approximately three fold. Instead, with a charge of 2.0 F the yield of **2a** improved by 10%, but the yield of **2b** increased only 2% (Table 2, entry 3). However, in these experiments methyl ester **3a** was isolated (24% yield) as byproduct derived from methanol, which passed through the membrane, reacting with the Breslow intermediate through a redox neutral process (Scheme 5) [19].

We decided to use 1 F/mol and a lower amount of methanol at the anode, in order to minimize the formation of byproduct **3a**; the same amount of **2a** was obtained, but the yield of the *trans* diastereoisomer increased from 24% to 32% (Table 2, entry 2 vs entry 4). Finally, changing methanol with DMSO in the anolyte, a lower amount of both diastereoisomers was observed (Table 2, entries 5 and 6). Thus the best yield (79%, 67:33 *cis*/*trans* ratio) was obtained using a charge of 2.0 F and a solution of MeCN–MeOH (9.5:0.5)/Et_4_NBF_4_ (0.1 M) as anolyte (Table 2, entry 3).

### Electrogenerated NHC organocatalysis. Esterification of *trans* cinnamaldehyde

Once the possibility of obtaining the Breslow intermediate was demonstrated, another typical reaction of N-heterocyclic carbenes was tested: the oxidative esterification of cinnamaldehyde in the presence of alcohols. To carry out these experiments three different alcohols (methyl, isopropyl and benzyl alcohols) were used and the results are shown in Table 3.

**Table 3:** Electrochemical synthesis of esters **3a–c** from cinnamaldehyde and an alcohol.^a^

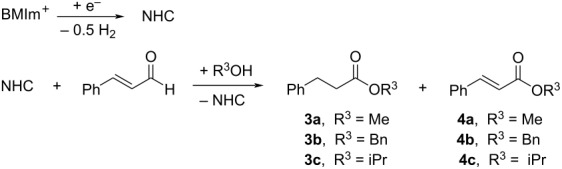					

Entry	ROH	Anolyte	*Q* (F)^b^	Yield^c^	

1	R^3^ = Me	MeCN–MeOH (98:2)/Et_4_NBF_4_ 0.1 M, 20 mL	0.5	**3a** 68%	**4a** 11%
2	R^3^ = Bn	MeCN–:BnOH (98:2)/Et_4_NBF_4_ 0.1 M, 20 mL	0.5	**3b** 73%	**4b** 13%
3	R^3^ = iPr	MeCN–iPrOH (98:2)/Et_4_NBF_4_ 0.1 M, 20 mL	0.5	**3c** 37%	**4c** –

^a^Divided cell, carbon-filled polyvinylidene fluoride (C/PVDF) anode, stainless steel cathode, alkaline pretreated Nafion^®^ 438 membrane separator, room temperature, N_2_ atmosphere, galvanostatic conditions (134 mA), catholyte: BMImBF_4_/MeCN 0.1 M, 20 mL (2 mmol BMImBF_4_); flow rate: 36 mL/min; 1 mmol of cinnamaldehyde added at the end of the electrolysis to the catholyte and, after 5 minutes 2 mmol of the corresponding alcohol were added. ^b^With respect to the starting BMImBF_4_, *t*_electrolysis_ = 12 min. ^c^Isolated yields, based on starting cinnamaldehyde.

All the experiments were carried out using a solution of 0.1 M of BMImBF_4_ in acetonitrile (20 mL) as catholyte, stainless steel as cathode, C/PVDF as anode, in a divided cell, under N_2_ atmosphere, at room temperature, under galvanostatic conditions (*I* = 134 mA, *t*_electrolysis_ = 12 min) with a flow rate of 36 mL/min, anode solution as in Table 3, and with 1.0 Faraday per mole of aldehyde. At the end of the electrolysis, 1 mmol of cinnamaldehyde was added to the catholyte and the mixture was left under stirring for five minutes and then the corresponding alcohol was added and the reaction was stirred for two hours at room temperature. Workup and column chromatography yielded esters **3a–c** and unsaturated esters **4a**,**b** as byproducts.

Good yields were obtained using benzyl and methyl alcohols (73% and 68%, respectively), while with the isopropyl alcohol the formation of the ester was only 37%, probably due to steric hindrance. In two cases (Table 3, entries 1 and 2) oxidation byproducts (esters **4a** and **4b**) were obtained, where the olefinic double bond is preserved. In contrast to the internal redox reactions of cinnamaldehyde giving esters **3a–c**, formation of enoate byproducts **4a** and **4b** invoke the involvement of an external chemical oxidant species as cinnamaldehyde is added to the cathode chamber after the electrolysis has been stopped. The presence of byproducts **4a** and **4b** is most likely accounted for by the presence of molecular oxygen, or electrochemically generated superoxide (cathodic reduction of O_2_), which oxidize the Breslow intermediate [30,39]. In fact, the presence of some reactive oxygen species in the reaction environment was previously demonstrated by the formation of compound **1b** (see Table 1).

### IL Recycling

To investigate the possibility to recycle BMImBF_4_, the experiment reported in Table 3, entry 1, was replicated, using the same reagents and conditions. Exploiting the low vapor pressure of BMImBF_4_, (and of ILs in general), the organic solvent was removed under reduced pressure, and after the extractive workup, the recycled BMImBF_4_ was resubjected to cathodic reduction (thus reused in different reactions). Although this procedure led to decreased yields of esterification products **3a**, **4a** (10% and 12%, respectively) and diastereoisomeric 4-phenyl-5-styryldihydrofuran-2(3*H*)-ones **2a**,**b** (35%, 45:55 *cis*/*trans* mixture), the potential of the recycled BMImBF_4_ to function as a N-heterocyclic carbene precursor was demonstrated. Interestingly, the reaction selectivity is completely lost using the recovered IL, and experiments are undergoing in order to understand the cause of this selectivity loss.

### Batch vs flow electrolysis

At this point, the preliminary results from flow electrochemistry may be compared with those reported in the literature for NHC formation using batch electrolysis. The comparison between these two techniques is not straightforward. In fact, in batch electrosynthesis, the ionic liquid is normally used as solvent, but in this work the IL was present as a 0.1 M solution in MeCN.

In regard to the results obtained in the synthesis of thione **1a** (the only reaction for which a reasonable comparison is possible), we compared the current efficiencies, the current being the limiting factor (Table 4). Although the current yield (49%) for the batch electrolysis in pure IL is higher than in flow, the yields for 0.1 M solutions are comparable between batch (29%) and flow (32%). In this case, the rate of production of the NHC in the electrolysis step was higher in the flow reactor (1.60 mmol/h) than in batch using pure IL (0.37 mmol/h). However, other factors should also be highlighted, including the 8× larger electrode area in the flow reactor, which enables a higher current to be passed while maintaining the same current density (15 mA/cm^2^).

**Table 4:** Comparison between electrosynthesis in batch and flow electrochemistry.

Product	Solvent	Batch electrochemistry yield (current density)	Flow electrochemistry yield (current density)

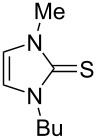 **1a**	BMImBF4	49%a (15 mA/cm2)	–
	solvent/BMImBF4	29%b (15 mA/cm2)	32%c (15 mA/cm2)
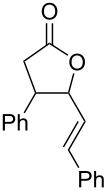 **2a,b**	MeCN/BMImBF4	–	79%d (15 mA/cm2)
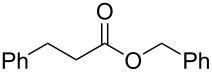 **3a**	BMImBF4	91%e (20 mA/cm2)	–
	MeCN/BMImBF4	–	73%f (15 mA/cm2)

^a^Current yield. *Q* = 193 C. Theoretical current for a mono-electron process: 96.5 C for 1.0 mmol substrate/product. Current yield: (experimental yield/ theoretical yield) × 100. [38]. ^b^Current yield (see note a). DMF as solvent. [12]. ^c^Current yield (see note a). MeCN as solvent; flow rate: 36 mL/min. ^d^Chemical yield, with respect to starting cinnamaldehyde. 2.0 F/mol cinnamaldehyde; flow rate: 36 mL/min. ^e^Chemical yield, with respect to starting cinnamaldehyde. 0.7 F/mol cinnamaldehyde. 0.97 mmol/h [40]. ^f^Chemical yield, with respect to starting cinnamaldehyde. 3.65 mmol/h. 1.0 F/mol cinnamaldehyde; flow rate: 36 mL/min.

In the case of the NHC organocatalysis, we compared chemical yields of products (Table 4), although in different solvents. As far as the literature allows comparison of the two methodologies, the data in Table 4 show that, although further optimization of the flow electrochemistry conditions is required, it provides a valid alternative to batch technique. Again, productivity for the esterification is four-fold higher (3.65 mmol/h, 0.1 M solution) in flow than in batch (0.97 mmol/h, pure IL), and in flow the reaction volume could simply be increased to allow scale-up, obviously in a longer time.

## Conclusion

The results reported in this work demonstrate for the first time the possibility to synthesize and accumulate N-heterocyclic carbene starting from BMImBF_4_ in a divided electrochemical flow cell. Although not fully optimized, the production of NHC was confirmed indirectly by isolation of its reaction product with elemental sulfur. A solution of the electrogenerated carbene was used to promote dimerization and oxidative esterification reactions of cinnamaldehyde. Under the flow conditions investigated, a higher rate of NHC production was achieved, compared to previously reported batch reactions, which is an important consideration for scale up. In the case of the flow procedure, a 0.1 M solution of the IL is employed, which may be more convenient than using pure ILs. Further investigations of electrogeneration of NHCs and applications in organic synthesis are underway.

## Experimental

### Materials and methods

Chemicals were purchased from Sigma-Aldrich and Alfa Aesar and used as received. All air/moisture sensitive reactions were carried out under an inert atmosphere, in oven-dried or flame-dried glassware. TLC was performed on aluminium plates precoated with silica gel 60 with an F_254_ indicator; visualized under UV light (254 nm) and/or by staining with potassium permanganate. Flash column chromatography was performed using high purity silica gel, pore size 60 Å, 230–400 mesh particle size, purchased from Merck. ^1^H NMR and ^13^C NMR spectra were recorded in CDCl_3_ (purchased from Cambridge Isotope Laboratories) at 298 K using a Bruker DPX400 (400 and 101 MHz, respectively) spectrometer. Chemical shifts are reported on the δ scale in ppm and were referenced to residual solvent (CDCl_3_: 7.27 ppm for ^1^H NMR spectra and 77.0 ppm for ^13^C NMR spectra). Coupling constants (*J*) were given in Hz and matched where possible. The following abbreviations for the multiplicity of the peaks are used: s (singlet), d (doublet), t (triplet), q (quartet) and m (multiplet).

### Parallel plate divided flow cell

**Figure 3:**
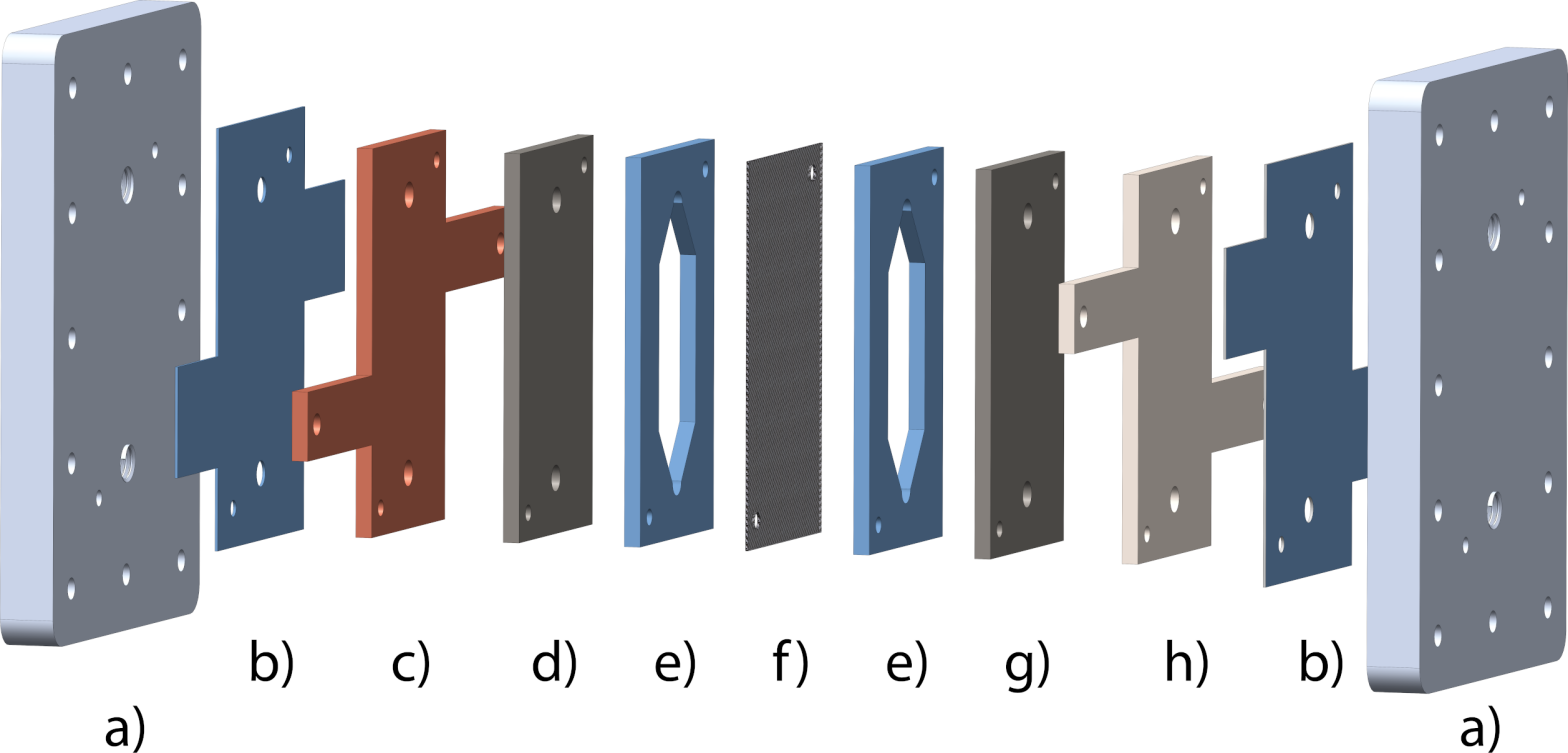
Expanded view of the electrochemical cell components: (a) Aluminium end plates; (b) insulating PTFE foil, (c) copper plate for electrical contact; (d) carbon electrode; (e) PTFE gasket (reaction channel and sealing); (f) Nafion^®^ membrane; (g) stainless steel electrode; (h) stainless-steel plate/electrode for electrical contact. Figure 3 was adapted from [36] with permission from The Royal Society of Chemistry. This content is not subject to CC BY 4.0.

See Figure 3 for an expanded view of the electrochemical cell components. The details about the dimensions of each cell component are reported elsewhere [36].

### General procedure for the synthesis of 1-butyl-3-methyl-1*H*-imidazole-2(3*H*)-thione

Constant current electrolyzes (*I* = 134 mA) were carried out using a parallel plate divided cell. Anolyte (25 mL) and catholyte (20 mL) were separated through a Nafion^®^ 438 membrane. The anode material was carbon-filled polyvinylidene fluoride (C/PVDF) and the cathode material is described in Table 1. Electrolyzes were carried out at room temperature, under nitrogen atmosphere, using a solution of 0.1 M of BMImBF_4_ in acetonitrile as catholyte. Anolyte solution is given in Table 1. Electrolyte solutions were continuously passed (recycling method) through their respective compartments at a flow rate of 36 mL/min. After consumption of the requisite amount of charge (Faradays per mol of BMImBF_4_ reported in Table 1), the current was switched off, the flow stopped, and elemental sulfur (2.0 mmol) was added to the catholyte. The mixture was left under ultrasound irradiation for 30 minutes. The solvent was removed under reduced pressure and the residue was purified by column chromatography on silica gel (petroleum ether/ethyl acetate 7:3), affording the corresponding pure **1a** as an off-white solid.

**1-Butyl-3-methyl-1*H-*imidazole-2(3*H*)-thione (1a):** Spectral data are consistent with those reported in the literature [38]. ^1^H NMR (CDCl_3_) δ 6.64 (s, 2H), 3.99 (t, *J* = 7.4 Hz, 2H), 3.57 (s, 3H), 1.79–1.64 (m, 2H), 1.39–1.28 (m, 2H), 0.92 (t, *J* = 7.3 Hz, 3H) ppm; ^13^C NMR (CDCl_3_) δ 162.3, 117.5, 116.4, 47.8, 35.0, 30.9, 19.7, 13.6 ppm.

**1-Butyl-3-methyl-1*H*-imidazol-2(3*H*)-one (1b):** Spectral data are consistent with those reported in the literature [40]. ^1^H NMR (CDCl_3_) δ 6.19 (d, AB, Δν/*J* = 2.5, Δν = 7.1 Hz, *J* = 2.8 Hz, 1H), 6.17 (d, AB, Δν/*J* = 2.5, Δν = 7.1 Hz, *J* = 2.8 Hz, 1H), 3.59 (t, *J* = 7.2 Hz, 2H), 3.24 (s, 3H), 1.70–1.59 (m, 2H), 1.42–1.24 (m, 2H), 0.92 (t, *J* = 7.2 Hz, 3H) ppm; ^13^C NMR (CDCl_3_) δ 153.1, 111.3, 110.2, 43.5, 31.1, 30.5, 19.9, 13.6 ppm.

### General procedure for dimerization of *trans* cinnamaldehyde

Constant current electrolyzes (*I* = 134 mA) were carried out using a parallel plate divided cell. Anolyte (20 mL) and catholyte (20 mL) were separated through a Nafion^®^ 438 membrane. The anode material was carbon-filled polyvinylidene fluoride C/PVDF and stainless steel for the cathode material. Electrolyzes were carried out at room temperature, under nitrogen atmosphere, using a solution 0.1 M of BMImBF_4_ in acetonitrile as catholyte and for anolyte solution see Table 2, which were passed (recycling method) in their compartments with a flow rate of 36 mL/min. After the consumption of the number of Faradays per mol of BMImBF_4_ reported in Table 2, the current was switched off, the flow stopped and cinnamaldehyde (1.0 mmol) was added to the catholyte. The mixture was stirred for 2 h. The solvent was removed under reduced pressure and the residue was extracted with of Et_2_O (15 mL × 3) and then purified by column chromatography on silica gel (petroleum ether/ethyl acetate 9:1), affording the corresponding pure products **2a** and **2b**.

**(±) *cis*-4-Phenyl-5-((*E*)-styryl)dihydrofuran-2(3*H*)-one (2a):** Spectral data are consistent with those reported in the literature [41]. ^1^H NMR (CDCl_3_) δ 7.38–7.25 (m, 3H), 7.29–7.24 (m, 3H), 7.23–7.15 (m, 4H), 6.63 (d, *J* = 15.9 Hz, 1H), 5.65 (dd, *J* = 15.9 Hz, 6.5 Hz, 1H), 5.42–5.38 (m, 1H), 3.99–3.93 (m, 1H), 2.98 (dd, *J* = 17.4 Hz, 8.1 Hz, 1H), 2.91 (dd, *J* = 17.4 Hz, 7.4 Hz, 1H) ppm; ^13^C NMR (CDCl_3_) δ 176.3, 136.9, 135.8, 133.4, 128.9, 128.6, 128.2, 127.9, 127.8, 126.6, 123.8, 83.5, 45.6, 34.3 ppm.

**(±) *trans*-4-Phenyl-5-((*E*)-styryl)dihydrofuran-2(3*H*)-one (2b):** Spectral data are consistent with those reported in the literature [41]. ^1^H NMR (CDCl_3_) δ 7.39–7.27 (m, 10H), 6.60 (d, *J* = 15.9 Hz, 1H), 6.23 (dd, *J* = 15.9 Hz, 6.7 Hz, 1H), 5.06–5.02 (m, 1H), 3.57–3.50 (m, 1H), 3.04 (dd, *J* = 17.5 Hz, 8.6 Hz, 1H), 2.87 (dd, *J* = 17.5 Hz, 10.5 Hz, 1H) ppm; ^13^C NMR (CDCl_3_) δ 175.2, 138.0, 135.6, 133.8, 129.2, 128.7, 128.4, 127.9, 127.3, 126.8, 124.7, 86.7, 48.4, 36.7 ppm.

### General procedure for the esterification of *trans*-cinnamaldehyde

Constant current electrolyzes (*I* = 134 mA) were carried out using a parallel plates divided cell. Anolyte (20 mL) and catholyte (20 mL) were separated through a Nafion^®^ 438 membrane. The anode material was C/PVDF and stainless steel for the cathode material. Electrolyzes were carried out at room temperature, under nitrogen atmosphere, using a solution 0.1 M of BMImBF_4_ in acetonitrile as catholyte. Anolyte solution is given in Table 3. Electrolyte solutions were continuously passed (recycling method) through their respective compartments at a flow rate of 36 mL/min. After the consumption of 0.5 F/mol of BMImBF_4_ (12 min, 96 C), the current was switched off, the flow stopped and cinnamaldehyde (1.0 mmol) was added to the catholyte. The mixture was left under stirring for 5 minutes and then the corresponding alcohol (2.0 mmol) was added and the reaction was stirred for 2 hours at room temperature. The solvent was removed under reduced pressure and the residue was extracted with of Et_2_O (15 mL × 3) and purified by column chromatography on silica gel (petroleum ether/ethyl acetate 9.5:0.5), affording the corresponding pure esters **3a–c** and **4a,b**.

### BMImBF_4_ Recycling procedure

After extraction of product **3a** from the solution, the catholyte was placed under vacuum at room temperature for 30 min to remove diethyl ether residues, then it was used as catholyte for a new electrolysis (see the general procedure for the esterification of *trans*-cinnamaldehyde to repeat the same procedure).

**Methyl 3-phenylpropanoate (3a):** Spectral data are consistent with those reported in the literature [42]. ^1^H NMR (CDCl_3_) δ 7.32–7.23 (m, 2H), 7.22–7.14 (m, 3H), 3.65 (s, 3H), 2.94 (t, *J* = 7.9 Hz, 2H), 2.62 (dd, *J* = 8.4 Hz, 7.3 Hz, 2H) ppm; ^13^C NMR (CDCl_3_) δ 173.3, 140.5, 128.5, 128.5, 128.3, 126.3, 51.6, 35.7, 30.9 ppm.

**Benzyl 3-phenylpropanoate (3b):** Spectral data are consistent with those reported in the literature [43]. ^1^H NMR (CDCl_3_) δ 7.19–7.39 (m, 10H), 5.13 (s, 2H), 2.99 (t, *J* = 7.0 Hz, 2H), 2.70 (t, *J* = 7.0 Hz, 2H) ppm; ^13^C NMR (CDCl_3_) δ 172.7, 140.4, 136.0, 128.6, 128.5, 128.3, 128.2, 126.3, 66.3, 35.9, 31.0 ppm.

**Isopropyl 3-phenylpropanoate (3c):** Spectral data are consistent with those reported in the literature [44]. ^1^H NMR (CDCl_3_) δ 7.28–7.36 (m, 2H), 7.19–7.27 (m, 3H), 4.99–5.11 (m, 1H), 2.99 (t, *J* = 7.5 Hz, 2H), 1.24 (d, *J* = 6.3 Hz, 6H) ppm; ^13^C NMR (75 MHz, CDCl_3_) δ 172.5, 140.6, 128.5, 128.4, 126.2, 67.8, 36.3, 31.1, 21.8 ppm.

**Methyl cinnamate (4a):** Spectral data are consistent with those reported in the literature [45]. ^1^H NMR (CDCl_3_) δ 7.69 (d, *J* = 16.0 Hz, 1H), 7.58–7.45 (m, 2H), 7.41–7.32 (m, 3H), 6.43 (d, *J* = 16.0 Hz, 1H), 3.78 (s, 3H) ppm; ^13^C NMR (CDCl_3_) δ 167.4, 144.8, 134.4, 130.3, 128.9, 128.1, 117.8, 51.7 ppm.

**Benzyl cinnamate (4b):** Spectral data are consistent with those reported in the literature [46]. ^1^H NMR (CDCl_3_) δ 7.73 (d, *J* = 16.0 Hz, 1H), 7.56–7.49 (m, 2H), 7.45–7.31 (m, 8H), 6.49 (d, *J* = 16.0 Hz, 1H), 5.26 (s, 2H) ppm; ^13^C NMR (CDCl_3_) δ 166.8, 145.2, 136.1, 136.0, 134.3, 130.4, 128.9, 128.6, 128.29, 128.26, 128.1, 117.9, 66.4 ppm.
